# Survival Analysis of Patients with Heart Failure: Implications of Time-Varying Regression Effects in Modeling Mortality

**DOI:** 10.1371/journal.pone.0037392

**Published:** 2012-06-08

**Authors:** Suely Ruiz Giolo, José Eduardo Krieger, Alfredo José Mansur, Alexandre Costa Pereira

**Affiliations:** 1 Laboratory of Genetics and Molecular Cardiology, Heart Institute, University of Sao Paulo, Sao Paulo, Sao Paulo, Brazil; 2 Department of Statistics, Federal University of Parana, Curitiba, Parana, Brazil; 3 Department of Clinical Cardiology, Heart Institute, University of Sao Paulo, Sao Paulo, Sao Paulo, Brazil; Sapienza University of Rome, Italy

## Abstract

**Background:**

Several models have been designed to predict survival of patients with heart failure. These, while available and widely used for both stratifying and deciding upon different treatment options on the individual level, have several limitations. Specifically, some clinical variables that may influence prognosis may have an influence that change over time. Statistical models that include such characteristic may help in evaluating prognosis. The aim of the present study was to analyze and quantify the impact of modeling heart failure survival allowing for covariates with time-varying effects known to be independent predictors of overall mortality in this clinical setting.

**Methodology:**

Survival data from an inception cohort of five hundred patients diagnosed with heart failure functional class III and IV between 2002 and 2004 and followed-up to 2006 were analyzed by using the proportional hazards Cox model and variations of the Cox’s model and also of the Aalen’s additive model.

**Principal Findings:**

One-hundred and eighty eight (188) patients died during follow-up. For patients under study, age, serum sodium, hemoglobin, serum creatinine, and left ventricular ejection fraction were significantly associated with mortality. Evidence of time-varying effect was suggested for the last three. Both high hemoglobin and high LV ejection fraction were associated with a reduced risk of dying with a stronger initial effect. High creatinine, associated with an increased risk of dying, also presented an initial stronger effect. The impact of age and sodium were constant over time.

**Conclusions:**

The current study points to the importance of evaluating covariates with time-varying effects in heart failure models. The analysis performed suggests that variations of Cox and Aalen models constitute a valuable tool for identifying these variables. The implementation of covariates with time-varying effects into heart failure prognostication models may reduce bias and increase the specificity of such models.

## Introduction

Patients with heart failure usually experience a progressive clinical deterioration over time. Factors that influence the unfavorable outcome are less predictable over time as they may be dependent on several, and distinct, factors such as pump failure, autonomic nervous system influence, cardiac arrhythmias, metabolic derangements (such as renal failure, hyperkalemia, hypokalemia), and complications that many times may be subclinical or undiagnosed, such as pulmonary embolism. This myriad of potential complications that may ensue in spite of current therapy are less predictable over time. Some of them, like progressive pump failure may be expected to have a more linear downhill course; others may not.

The incidence and prevalence of heart failure (HF) are rising worldwide [1]. And although decline trends in HF hospitalization rates have been shown in Europe [2] and in the USA [3], current advances in the treatment of both myocardial infarction and heart failure itself bring the forecast of even higher heart failure numbers. At the same time, new indications and care for the transplanted patient is continuously emerging and new ventricular assist-devices are yearly being introduced into clinical practice [4], [5]. This scenario has brought increasing interest in the development of new and more sensitive and specific tools for heart failure prognostication [6].

In fact, a number of different tools for heart failure prognostication already exist and are increasingly being incorporated into clinical practice [7], [8]. These include the Heart Failure Survival Score [9], the Seattle Heart Failure Model [10], the Organized Program to Initiate Lifesaving Treatment in Hospitalized Patients With Heart Failure predictive schemes [11], the Acute Decompensated Heart Failure National Registry regression tree discrimination [10], among others. Interestingly, they were built using rather different patient populations and analytical tools for model construction. Some were specifically designed for acute decompensated HF and were not built to be used with out-patient populations [11], [12]. These scores do not rely on survival analysis for their construction and use different data supposition for their validity, being specifically tailored for the hospitalized patient with HF. On the other hand, there are well-established tools for the out-patient scenario, all of them built in the outline, and constrains, of survival analysis [9], [10]. Although well-designed and validated, these models do not consider time-varying effects of their covariates and relied upon the framework of proportional hazards Cox regression, which assumes proportionality of the hazards and also that the risk factors act multiplicatively on the baseline hazard risk function. These assumptions, however, may not be proper in some applications and there is therefore the need for alternative models.

Risk factors may also have additive effects instead of multiplicative effects in the baseline hazard function. Another typical deviation from the proportional hazards Cox model is when the effects of some covariates change with time. For instance, some risk factors may impose a strong effect right after being recorded, but gradually lose predictive power (e.g. a treatment effect that is weakened with time). Models flexible enough to deal with covariates in which their effects are time-varying are therefore of great interest in these situations. One of these models is a direct extension of the proportional hazards Cox model where all or some effects of the covariates are allowed to change over time [13]. Another is the additive hazards model proposed by Aalen [14]–[16] that allows all regression coefficients to vary with time. As, however, the effect of some of the covariates may change with time while others not, McKeague and Sasieni [17] suggested a variation of the Aalen model allowing for this possibility.

Here we have used these models to analyze the survival of patients diagnosed with heart failure. Our main aim was to explore the time-varying effect of the different covariates known to be predictive of mortality in such clinical scenario and highlight the importance of considering such details in the modeling of heart failure mortality.

## Materials and Methods

### Ethics Statement

All patients signed an informed consent form and the study has been approved by the Ethics Committee of the Heart Institute of the University of Sao Paulo, Brazil.

### Study Sample

Five hundred (500) patients with heart failure in functional class III or IV of the New York Heart Association were studied. Patients were included as part of a secondary-cohort of HF individuals attended at a cardiology tertiary care center in Sao Paulo, Brazil (Heart Institute of the Sao Paulo University Medical School). Ascertainment period was from August, 2002 to March, 2004.

The diagnosis of heart failure was made according to previously published criteria [18], [19]. The classification of the etiologies of heart failure followed previous recommendations [19]. As such, the diagnosis of chronic heart failure was made through both clinical and imaging procedures when necessary. Ischemic cardiomyopathy diagnosis was made when a clear history of previous myocardial infarction and no other probable cause of heart dysfunction was present or, alternatively, through coronary angiography. All patients with the final diagnosis of idiopathic dilated cardiomyopathy were studied through coronary angiography to exclude the diagnosis of ischemic cardiomyopathy. Therapy was titrated according to the patient’s needs and tolerance by the physician in charge and included angiotensin-converting enzyme blockers, angiotensin receptor blockers, diuretics, and beta-blocker (carvedilol). Carvedilol is the standard beta-blocker prescribed at the Heart Institute of the Sao Paulo University. Spironolactone was used only in a very small fraction of patients (probably reflecting the enrollment period of the cohort).

Beginning of follow-up was defined as enrollment in the protocol. Follow-up was assessed in the last outpatient medical visit or by telephone contact. In addition, the mortality database of Sao Paulo City Authority was also scrutinized to discover patient deaths (ProAim − Programa de Aprimoramento de Informações de Mortalidade do Município de São Paulo). For the current analysis, last follow-up was evaluated in April, 2006. Primary end-point studied was overall mortality. Table 1 summarizes the descriptive information available.

**Table 1:** Clinical characteristics of the study sample.

Variables	Descriptive information
Age	18 to 93 yrs (mean = 58.08, sd = 14.38)
Gender	59% male (297) and 41% female (203)
Race	73.4% white (367) and 26.6% others (126)
Diabetes mellitus	25.8% (129)
Hypertension	63% (315)
Current smoking	9% (46)
BMI (body mass index)	14.33 to 46.13 kg/m^2^ (mean = 25.57, sd = 5.39)
Left ventricular ejection fraction	0.09 to 0.88 (mean = 0.4538, sd = 0.1866)
Left ventricular mass	73.88 to 835.50 g (mean = 252.1, sd = 94.16)
Serum sodium	117 to 147 mEq/L (mean = 136.7, sd = 4.52)
Hemoglobin (Hb)	6.6 to 14.6 g/dL (mean = 13.06, sd = 2.135)
Creatinine	0.6 to 10 mg/dL (mean = 1.35, sd = 0.74)
Etiology	12% Chagas (60), 28.6% Ischemic (143) and 59.4% other (297)

Other etiologies: idiopathic (n = 50), hypertensive (n = 143), valvular (n = 76) and other (n = 28); sd  =  standard deviation.

### Statistical Methodology

To describe the data, descriptive statistics were calculated (mean, median, standard deviation and frequencies) based on information available for the 500 patients in the study. Next, the covariates most probably associated with the survival time in days were investigated by using the Kaplan-Meier (KM) estimator [20]. Continuous covariates were, in general, considered in two categories based on their respective median values. The null hypothesis of no differences between the two survival curves for each covariate, i.e. H_0_: S_1_(*t*) = S_2_(*t*), was tested by logrank test [21].

Although the KM estimator is a useful descriptive tool, it has not been designed to incorporate several covariates simultaneously. Hence, to evaluate the effect of a risk factor on the survival adjusted for a set of other risk factors, we started by fitting the proportional hazards Cox model [22], in which the hazard function is modeled as
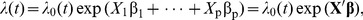
(1)where *λ_0_*(*t*) is an arbitrary baseline hazard rate, **X**  =  (X_1_, …, X_p_) are the *p* covariates or risk factors of interest, and **β**  =  (β_1_, …, β_p_)’ is a *p*-dimensional vector of regression coefficients which is estimated by considering the partial likelihood. Under this model, the hazard ratio is assumed constant over time (proportional hazards).

Covariates that change their values over time (time-dependent covariates), such as a dynamic treatment dose, can be included in model (1). For our data, however, covariates are not time-dependent since they were only measured at the beginning of the study. As the assumption of proportionality failed for some covariates, suggesting that the risk of a patient may change over time, even if their risk factors do not change, we next fitted an extension of model (1) to taken into account that may exist covariates with time-varying effects (i.e. non-proportional effects). Under this model the hazard function is expressed on the form
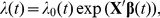
(2)where the vector of regression coefficients **β** has been replaced by **β**(*t*)  =  (β_1_(*t*), …, β_p_(*t*))’, which are functions representing the time-varying effects of covariates over time. Parameters estimates for this model can be obtained by considering the partial likelihood and by the choice of smoothing parameters [23]. As estimation of **β**(*t*) depends on smoothing methods, it is obtained by mathematical convenience the estimates of the cumulative time-varying effects, i.e. B_q_(t)  =  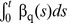 for *q*  = 1,…, *p*. The estimates of **β**(*t*) are thus the slopes of the cumulative estimates. Tests for whether the separate components of **B**(t)  =  (B_1_(*t*), …., B_p_(*t*))’are constant (i.e. H_0_: B_q_(*t*)  =  γ *t*), as well as tests for non-significant effects (H_0_: B_q_(*t*)  = 0), were then performed. Model (2) with some time-varying effects and others not is termed semi-parametric multiplicative hazards model and can be expressed as
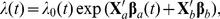
(3)where Xa and Xb represent the covariates with time-varying and constant effects, respectively. From the final model, estimates of the effects are provided and discussed. For those covariates with significant time-varying effects, their corresponding components of Ba(t) are shown graphically in terms of their cumulative regression estimates.

Even though model (2) or (3) appears very appealing, there are some drawbacks related to this model as, for instance, that it is hard to assess the uncertainty of the estimates β_q_(*t*) and also that it is not easy to estimate the corresponding survival function expressed as 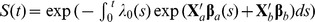 not only because we need to estimate **β**_a_(*t*) and **β**_b_ by means of smoothing methods, but also because it is complicated to work with the above integral [23]. To circumvent these difficulties, the Aalen’s additive hazards model [13]–[16] which allows covariates with time-varying effects was used. Under this model, the additive hazards are expressed as
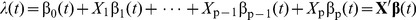
(4)where β_0_(*t*) represents the baseline hazard denoted by *λ_0_*(*t*) in the proportional hazards Cox model, **X**  =  (1, X_1_, …, X_p_) is a matrix containing a vector of ones and the *p* covariates (risk factors) of interest, and **β**(*t*)  =  (β_0_(*t*), β_1_(*t*),…,β_p_(*t*))’is a vector of time-varying regression coefficients. Covariates that change their values over time (time-dependent covariates) can also be considered in model (4). Similar to models (2) or (3), estimation and tests are based on the cumulative effects B_q_(t)  =  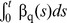 (*q*  = 1,…, *p*). For model (4), however, there are simple direct least squares estimators. Thus, it can be fitted without any use of smoothing parameters. To test if a covariate effect is time-varying or constant over time, we fitted a variation of model (4), termed semi-parametric additive hazards model, expressed as
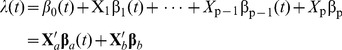
(5)where the effect of p −1 covariates change over time while the effect of one of them is assumed to be constant. The matrices Xa and Xb include the covariates with time-varying and constant effects, respectively. To test the null hypothesis of constant effect associated with the p-th covariate (H0: βp(t)  =  γ or equivalently H0: Bp(t)  =  γ t), models (4) and (5) were compared [13]. Successive tests were performed in this stage of the analysis until covariates with time-varying and constant effects were all characterized. For all covariates (q  = 1,…, p), tests for non-significant effects (H0: βq(t)  = 0 or equivalently H0: Bq(t)  = 0) were also performed. Procedures for these statistical tests are explained in details in Martinussen and Scheike [13]. From the final model, estimates of the constant and time-varying effects were then provided and discussed. Time-varying effects were shown graphically in terms of their cumulative regression functions estimates 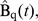
in which the slopes of their estimated functions represent the coefficients βq(t). Survival function expressed for this model as 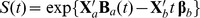 is much easier to obtain than under model (3) since it depends directly on B(t). Survival estimated curves were shown graphically for some patients in our study. The package survival available in the R software [24] was used to obtain the results for model (1). Results for models (2) and (3) were obtained in this same software using the packages timereg and coxvc [25], as well as for models (4) and (5) using the package timereg.

### Goodness-of-fit Procedures

To evaluate if the proportionality holds for each covariate in model (1), graphics and tests based on the scaled Schoenfeld residuals [26] were examined. No serious violation of the proportionality assumption is observed when these residuals plotted versus time for each covariate in the Cox model are randomly distributed around the zero-slope line. Graphics of the observed test-processes together with fifty simulated processes under proportionality were also investigated. The departure (deviation from the linear form) of the observed processes from the simulated curves under the model indicates those covariates having time-varying effects. Martinussen and Scheike [13] provide further details on these procedures. The Cox-Snell residuals [27] were also used as a way to check the overall fit of model (1). These residuals are defined as 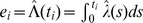 for *i*  = 1, …, *n,* and should look like a censored sample from a unit exponential distribution. For a model providing a satisfactory fit to the data, a plot of the survival probabilities of the residuals *e_i_*’s obtained by considering the unit exponential distribution 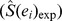 against those obtained by the Kaplan-Meier estimator 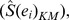 should be roughly a straight line through the origin with slope 1. Once a clear lack-of-fit of the proportional hazards Cox model was observed due to the effects of some covariates being strongly time-varying, violating the proportionality assumption, flexible alternatives to Cox model like models (2) and (4), or their semi-parametric versions (3) and (5), were used. Although additional studies are needed to investigate more appropriate methods to assess the goodness-of-fit of these models, here we have used procedures based on the cumulative martingale residuals [13] as a way to validate the fit of the models with time-varying effects to the data. Under these procedures, cumulative martingale residuals processes, that will carry information about the fit of the models as a function of each covariate, are plotted together with 50 simulated processes under the model for evaluating if their behavior is consistent with what should be expected under the model (zero-mean processes). A supremum test-statistic [13] was then computed to help summarize how serious can be a departure from the null processes.

## Results

### Descriptive Statistics

Table 1 summarizes the information available for patients that have been included in the study. Thirty-seven percent (37.6%) of the patients have died at the end of the follow-up period. From them, 62.8% were men and 37.2% women. Men accounted for 59.4 percent of the patients. The mean age observed was 58 years, with the range of 18 to 93 years. About 74% were white and 90.8% were non-smoking. The prevalence of diabetes and hypertension observed for all patients were 25.8% and 63%, respectively. The ischemic etiology was prevalent in relation to Chagas etiology (28.6% vs 12%) and overweight was predominant among women (50.3% vs 47%). For left ventricular ejection fraction the mean value was higher among women (0.51 for females against 0.41 for males). In opposite, the mean values for left ventricular mass, hemoglobin (Hb), and creatinine were higher among men; 277.5 vs 216.10 g, 13.47 vs 12.47 g/dL, and 1.44 vs 1.22 mg/dL, respectively. For serum sodium, the mean values were similar in both genders (136.6 mEq/L for males against 136.9 mEq/L for females).

In order to explore the variables usually recognized as influencing prognosis, survival curves for each covariate were estimated by using the Kaplan-Meier estimator. Differences between the curves were tested by logrank test. Categorization of continuous risk factors was done, in general, by considering two categories of the risk factor based on its median value. Table 2 displays the results of the tests performed. At a significance level of 5%, evidence of association with the time to death was suggested for seven of the thirteen covariates: age, body mass index (BMI), left ventricular ejection fraction, serum sodium, hemoglobin (Hb), creatinine, and etiology. It can also be noted that gender showed a marginally significant association.

**Table 2:** Logrank test performed for each covariate.

	Logrank test	
Covariates	Statistic	p-value
Age (≤60 and >60 yrs)	13.00	<0.001
Gender (male and female)	2.64	0.104
Race (white and others)	0.56	0.453
Diabetes mellitus (yes and no)	1.04	0.307
Hypertension (yes and no)	1.54	0.215
Current smoking (yes and no)	1.96	0.165
BMI (≤25 and >25 kg/m2)	6.16	0.013
LV ejection fraction (<0.35 and ≥0.35)	10.70	0.001
LV mass (≤243 and >243 g)	0.11	0.742
Serum sodium (≤137 and >137 mEq/L)	27.9	<0.001
Hemoglobin (Hb) (≤13 and >13 g/dL)	15.6	<0.001
Creatinine (≤1.2 and >1.2 mg/dL)	23.4	<0.001
Etiology (Chagas and others)	13.13	<0.001

Although the Kaplan-Meier estimator can always be used as a useful preliminary exploratory analysis, it is not proper to evaluate a risk factor effect adjusted for a set of other risk factors. Thus, to investigate the effect of each risk factor on time to death in the presence of a set of other risk factors, as well as the possibility of time-varying covariates effects, we next considered: (a) the proportional hazards Cox model, (b) an extension of the Cox’s model and (c) the additive hazards model to analyze the patients’ survival in our study. Before fitting these models, continuous covariates were centered on their respective mean values. For each of these models a selection strategy based on a forward stepwise approach with the use of a probability value of ≤0.05 for inclusion or deletion was used.

### Proportional Hazards Cox Model

For the proportional hazards Cox model, covariates (factors) showing significant effects were: age, serum sodium, hemoglobin (Hb), creatinine, and left ventricular ejection fraction. BMI, etiology and gender, suggested by the preliminary analysis as significant or marginally significant, were not significant in the presence of other factors in the model.

**Figure 1:**
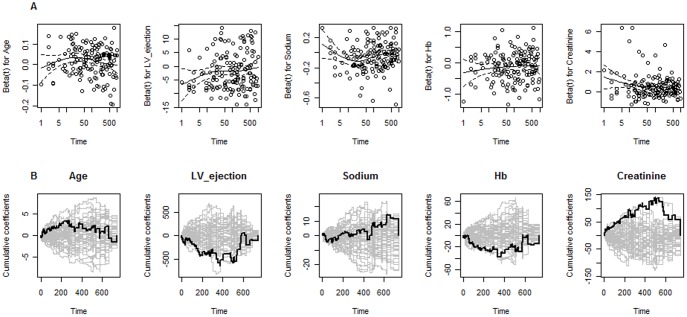
Graphical checks of the proportional hazards assumption. A. Scaled Schoenfeld residuals against time plotted for each covariate in the proportional hazards Cox model. B. Observed test process plotted along with 50 processes simulated for each covariate in the proportional hazards Cox model under the hypothesis of time-invariant effects.

For each covariate in the Cox model, Figure 1A displays the scaled Schoenfeld residuals versus time, together with a smooth scatter plot. The figure demonstrates evidence of deviation from the proportionality assumption since the plotted curves are not roughly constant over time for some covariates. Tests based on the scaled Schoenfeld residuals shown in Table 3 also suggest departures from the proportionality. Moreover, the observed test-process displayed in Figure 1B for each covariate along with 50 simulated processes under the null hypothesis of time-invariant effects, also suggest some covariates with no constant effects over time (hemoglobin, creatinine, and left ventricular ejection fraction). Hence, we may conclude that there is evidence of a lack-of-fit of the proportional hazards Cox model due to the effects of some covariates being time-varying.

**Table 3:** Test for the proportionality in the Cox model.

Covariates	Statistic	p-value
Age	1.39	0.24
Serum sodium	0.47	0.49
Hemoglobin	1.08	0.30
Creatinine	3.88	0.05^a^
LV ejection	2.99	0.08^a^

a Departures from the proportionality suggested for these covariates.

Tests based on the scaled Schoenfeld residuals.

### Variation of Cox Model Allowing Time-varying Covariates Effects

**Figure 2:**
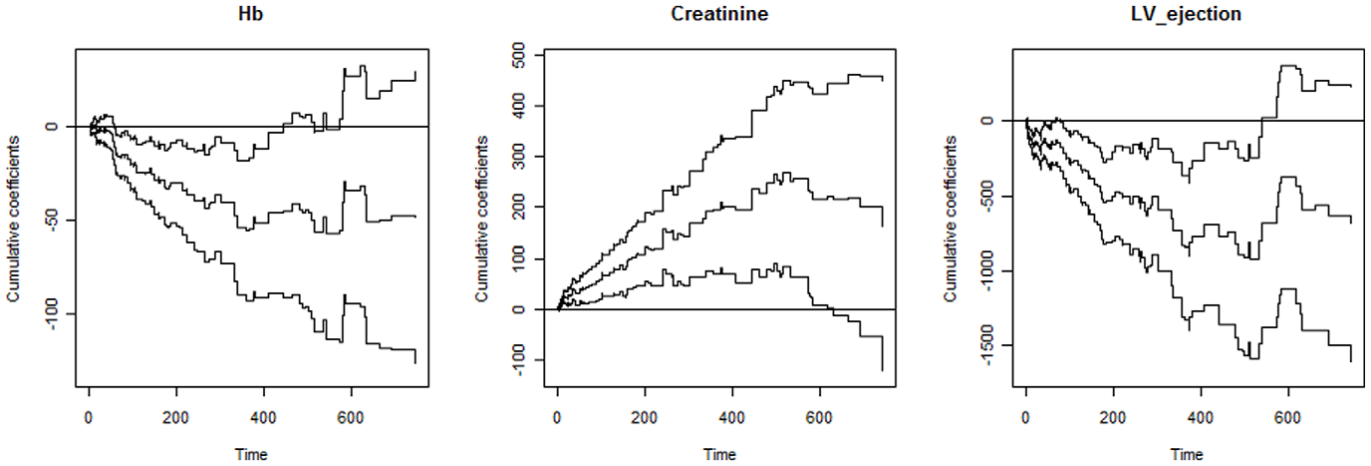
Cumulative coefficients obtained from the extended Cox’s model. Estimates from 1 to 750 days are for the covariates considered in the model as having time-varying effects. Curves along with the estimates are 95% confidence limits.

Under this model, significant effects were found for the same covariates as in the proportional hazards Cox model, i.e. age, serum sodium, Hb, creatinine, and left ventricular ejection fraction. Evidence of time-varying effects was indicated for hemoglobin (Hb), creatinine, and left ventricular ejection fraction as can be seen from Figure 1B. From such figure it is relatively easy to see departure from the zero line during the time-period for these covariates. Creatinine, for instance, has an effect which increases with time. From this same figure we can also see that the age and serum sodium covariates are characterized by their time-invariant effect since no pronounced departure from the zero line is observed (p-values of 0.72 and 0.43, respectively). Estimates of the effects of age and serum sodium were 0.027 (s.e.  = 0.00036) and −0.062 (s.e.  = 0.0018), respectively. Time-varying effects for hemoglobin, creatinine, and left ventricular ejection fraction covariates are shown in terms of their cumulative regression estimates in Figure 2.

### Additive Hazards Model

**Figure 3:**
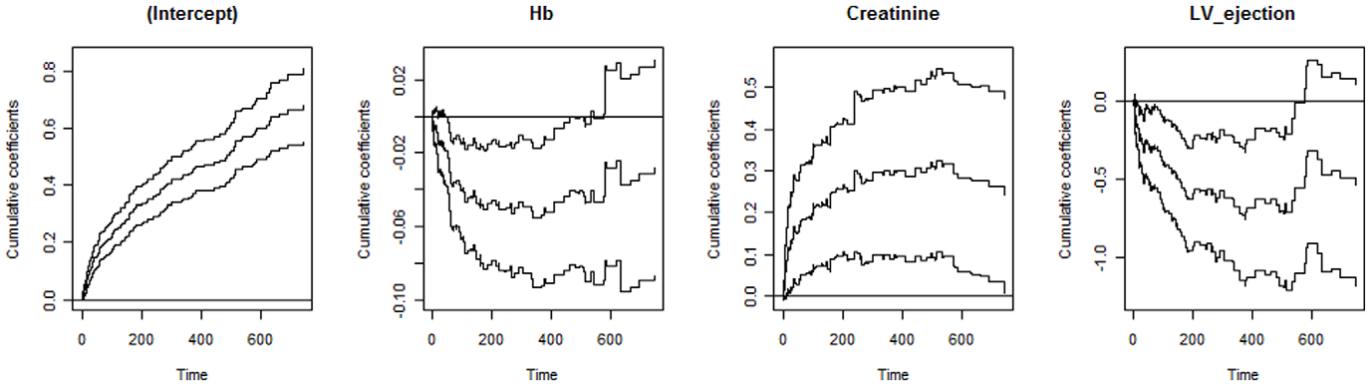
Cumulative coefficients obtained from the additive hazards model. Estimates from 1 to 750 days are for the covariates considered in the model as having time-varying effects. Curves along with the estimates are 95% confidence limits.

The covariates (factors) showing significant effects in the final additive hazards model were also: age, serum sodium, Hb, creatinine, and left ventricular ejection fraction. Evidence of time-varying effects was indicated for three of the selected factors (Hb, creatinine, and left ventricular ejection fraction). In agreement with the Cox model with time-varying effects, the impact of age and serum sodium was characterized by their constant effects over time. Note from Figure 3 that the cumulative regression coefficients associated with factors that were identified as having time-varying effects seem to change with time given that the cumulative does not seem to be a straight line as should be expected in case of time-invariant effects. The intercept curve corresponds to the cumulative hazard function for a patient with mean values of Hb, creatinine and LV ejection fraction. Table 4 displays the results of some tests related to the effects remaining in the final model. From them, it is possible to see that the covariates age and serum sodium showed time-invariant effects (p-values >0.15). Estimates of their effects were 1.6e^−5^ (s.e.  = 6.1e^−6^) and −8.5e^−5^ (s.e.  = 2.7e^−5^), respectively.

**Table 4:** Tests associated with the additive hazards model.

	Test for non-significant effect		Test for time-invariant effects	
Covariates	Statistics	p-value	Statistics	p-value
Intercept	10.70	<0.001	0.162	0.001
Age	2.85	0.048	0.004	0.291^a^
Serum sodium	3.33	0.018	0.017	0.199^a^
Hemoglobin	3.27	0.026	0.044	0.013
Creatinine	3.43	0.013	0.221	0.003
LV ejection fraction	3.88	0.001	0.502	0.007

a Time-invariant effects suggested for age and serum sodium (p>0.05).

All covariates were centered in their respective mean values.

### Checking the Goodness-of-fit of the Models

**Figure 4:**
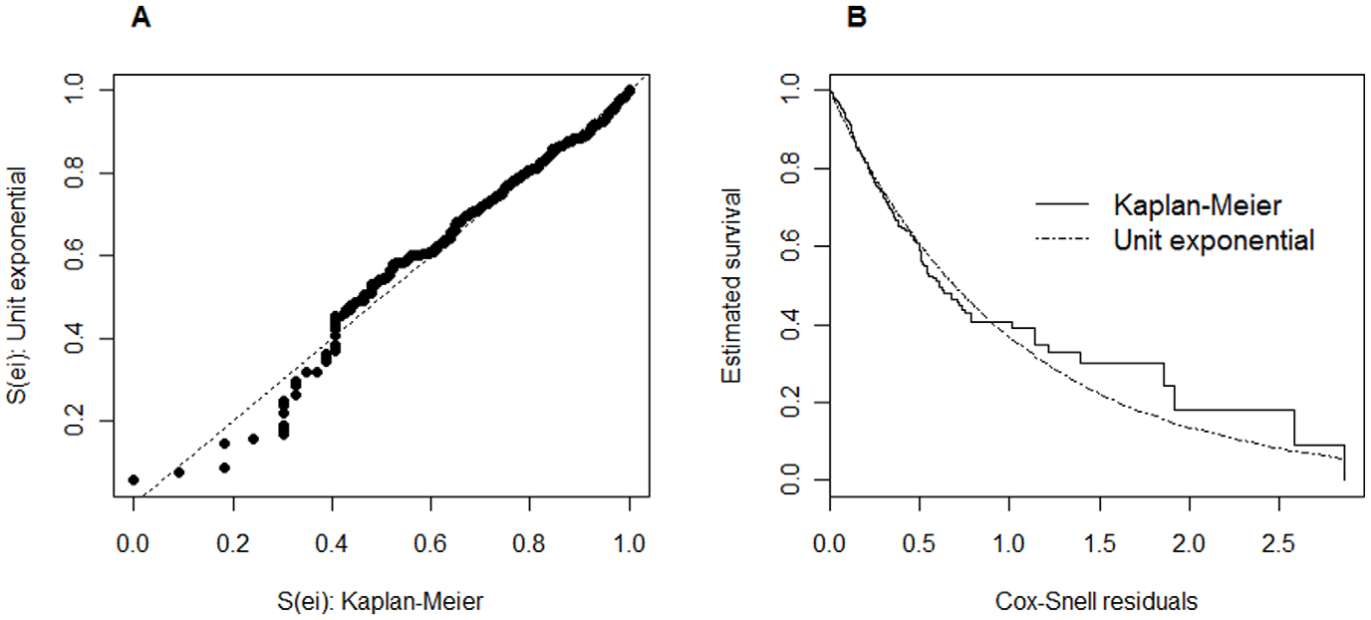
Graphical checks of the overall fit of the Cox model. A. Survival probabilities obtained from the Cox-Snell residuals by considering the unit exponential distribution and the Kaplan-Meier estimator. B. Survival curves obtained from the Cox-Snell residuals by considering the Kaplan-Meier estimator and the unit exponential distribution.

**Figure 5:**
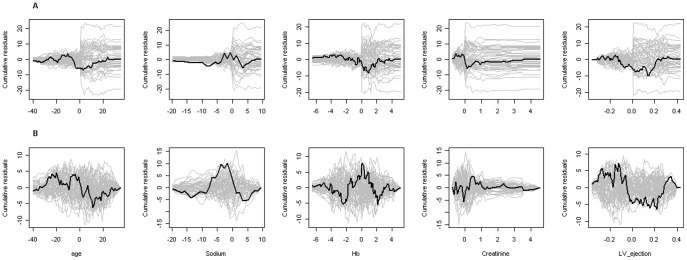
Cumulative martingale residuals plotted for each covariate. A. Cumulative residuals from the Cox́s model with time-varying effects. B. Cumulative residuals from the additive hazards model with time-varying effects.

**Figure 6:**
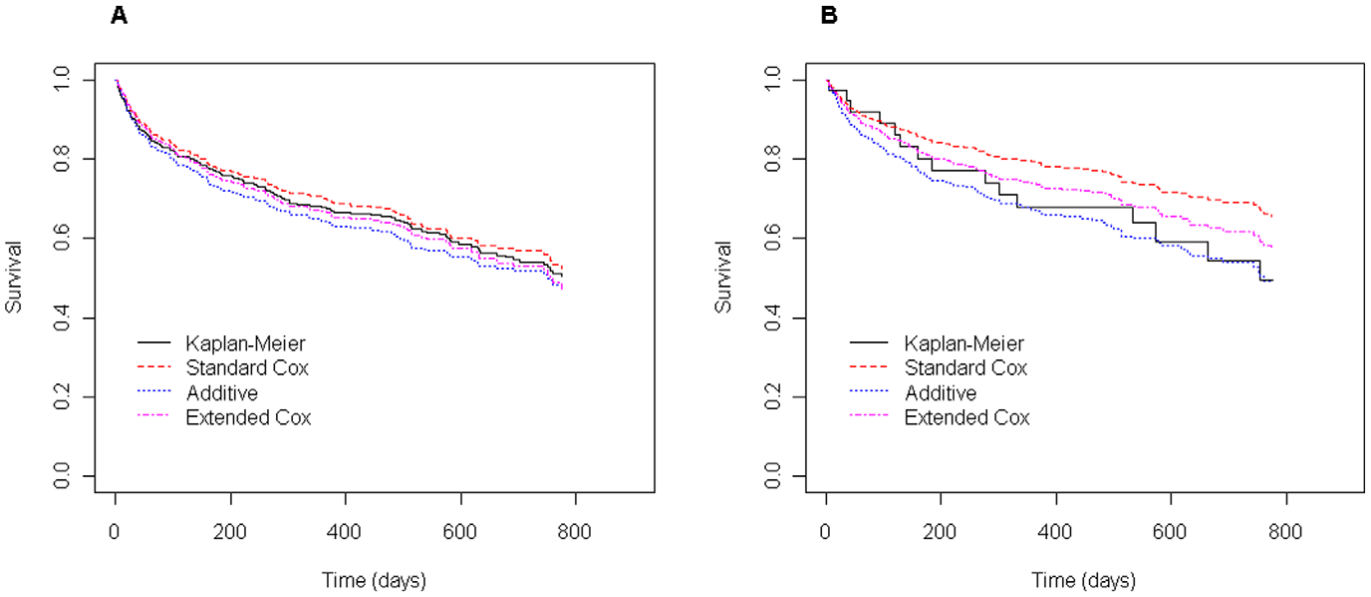
Non-parametric and model-based survival curves for two scenarios. A. Curves predicted for patients with mean values for all covariates (mean values in Table 1). B. Curves predicted for hypothetical patients aged 32 yrs old, serum sodium  = 137, Hb  = 13.7, creatinine  = 1.0 and LV ejection fraction  = 0.37. A subset of 39 patients who provided mean values equal to those considered in this scenario was used to estimate the Kaplan-Meier curve.

In order to assess if the fitted models provide an adequate fit to the data, one can use the Cox-Snell residuals for the proportional hazards Cox model and procedures based on martingale residuals [13] for the two time-varying regression models. From graphical analysis of the Cox-Snell residuals displayed in Figures 4A and 4B a moderate deviation from the unit exponential distribution can be observed, indicating that the Cox model presents a not too adequate fit to the data. On the other hand, the cumulative martingale residuals displayed in Figure 5A together with 50 simulated processes under the extended Cox model, suggest that all covariates have a behavior consistent with this model (zero-mean martingales). This is supported by the p-values of the supremum test-statistic shown in Table 5. Similar plots displayed in Figure 5B for the semi-parametric additive model indicate that the behavior of the residuals for the covariate sodium is not too consistent with this model (also supported by the supremum test-statistic shown in Table 5). In Figure 6 one can directly compare the different survival curves predicted from the models for two distinct clinical scenarios. In addition, a direct comparison with empirical data can be obtained by comparing the survival curves predicted from the models with the non-parametric Kaplan-Meier curves. Under the first clinical scenario (Figure 6A), no serious discrepancies can be observed between the non-parametric and model-based survival curves. But, in the second scenario (Figure 6B) survival predictions obtained particularly from the Cox model are quite different from those obtained by the Kaplan-Meier estimator. Although the considered models have difficulty to accommodate the heart failure dataset well, the additive modeling suggests to be slight better than the standard Cox and the extended Cox ones (Figure 6B), but none of them fit the data very well. Since from the 500 patients under study 188 died during the follow up, a possibility that one could think to enrich or enhance the overall goodness of fit is to consider models that accommodate the presence of long-term survivals. In this case, however, an aspect that must be taken into consideration is whether follow up in the data is sufficient [28]. In our study a longer follow up seems to be recommended since at the last follow-up time there were about 30% of patients with less than five years of follow-up and also about 15% who did not return.

**Table 5:** Tests for assessing covariates consistent with the extended models.

	Extended Cox model		Extended Additive model	
Covariates	Sup |hat B(t)|	p-value	sup|hat B(t)|	p-value
Age	5.34	0.87	6.14	0.61
Serum sodium	7.20	0.59	10.24	0.06
Hemoglobin	6.01	0.79	7.79	0.22
Creatinine	4.53	0.91	5.90	0.67
LV ejection	9.39	0.41	7.28	0.33

Supremum test-statistic was based on the cumulative martingale residuals.

## Discussion

Studies where the response is the time from a well defined moment in time to the occurrence of some event of interest are usual in many research areas. For handling these kinds of data several methods have been proposed in the last decades. Amongst them, are most known the non-parametric estimator proposed by Kaplan and Meier [20] and the proportional hazards Cox model [22], which assumes proportionality of the hazards. This assumption, however, may not be proper in some applications and there is the need for alternative models consider time-varying effects of their covariates.

Interestingly, from analyses performed in this paper, particularly on the scaled Schoenfeld residuals associated to the proportional hazards Cox model, we found evidence that suggested three of the most important predictors of outcomes in patients with heart failure (hemoglobin, creatinine and left ventricular ejection fraction) as having time-varying effects (Figures 1A, 1B and Table 3), meaning that the effects of such covariates are probably not constant over time, violating thus the assumption of proportional hazards. Although the time-varying effects observed in this cohort might not hold true in other populations, this brings into discussion whether scores derived from a Cox model framework without time-varying effects will be able to describe noticeable and important features of the data sufficiently well, or, put into a different perspective, whether using a Cox proportional hazards framework is the most competitive approach to derive proxy data that emulate the clinical scenario of heart failure, and that may be used for clinical prognostication and important treatment decisions.

In order to take into account time-varying effects, here we have used flexible variations of the Cox model and also of the Aalen additive hazards model in which some of the covariates are allowed to change over time while others not. These models provide alternative summary measures of the data, especially when the follow-up period is long, as in the present study, or in cancer studies, since part of the observed rate λ(t) is explained by the natural mortality of the background population. The covariates considered were only the best predictors of mortality in patients with heart failure, but the use of other predictors can be incorporated in future models.

Although methodological limitations have been found in this work with regard to appropriate methods for assessing the goodness of fit of the models evaluated, the results of our analyses were able to suggest that models do offer rather different survival estimates that could, in a clinical setting, provide significantly different thresholds for very expensive treatment options like heart transplantation, ventricular assist-device implantation, or ICD use. Predicted survival derived from these models are specially discordant from the proportional hazards Cox model (but not necessarily from empirically observed mortality) when one is more concerned with long follow-up times (see Figures 5 and 6), and specially for subgroups of patients that have values for time-varying variables that are distant from the mean values observed in the sample used for model derivation (see Figures 6A and 6B).

Clinical predictions (prognostic assessments) are usually based on a patient́s status (covariates) at the time of evaluation. Although there are alternatives to model covariate’s change over time or even to recalculate scores after changes in clinical status or medications/devices are prescribed, it isn’t always practical to take historical or future values of some covariates into account. The use of the described models may permit that, at baseline, one can account for the greater tendency of particular covariates to dynamically change.

Although we have graphically suggested better fit of the time-varying effects models over the standard Cox model, one of the limitations of the present work is the lack of validated analytical procedures to compare these different models in terms of their overall prediction capacity. In the paper we have used procedures based on the Cox-Snell and martingale residuals as a way to show that the models with time-varying effects can produce better goodness-of-fit than the proportional hazards Cox model since this model did not capture all important aspects of the data analyzed. One such alternative could perhaps be the Bayesian information criterion (BIC), but although in the proportional hazards Cox model, Volinsky and Raftery [29] propose defining BIC in terms of the maximized partial likelihood using the number of deaths rather than the number of individuals in the BIC penalty term, BIC has not yet been addressed for survival models with time-varying covariate effects like those used in this paper for analyzing the heart failure data. Therefore, additional studies are needed to investigate more appropriate methods to assess the goodness-of-fit of these models. Indeed, we consider this to be an analytical problem that should deserve more attention.

In conclusion, the analyses performed suggest that the extended Cox model and also variations of the additive hazards model are valuable tools for identifying covariates with time-varying effects present in the heart failure models. The implementation of time-varying covariate effects into heart failure prognostication models may reduce bias and increase the specificity of such models, thus contributing to more cost-effective management of patients with such condition.

## References

[1] Bleumink GS, Knetsch AM, Sturkenboom MC, Straus SM, Hofman A (2004). Quantifying the heart failure epidemic: prevalence, incidence rate, lifetime risk and prognosis of heart failure The Rotterdam Study. *Eur Heart J.*.

[2] Jhund PS, MacIntyre K, Simpson CR, Lewsey JD, Stewart S (2009). Long-Term trends in first hospitalization for heart failure and subsequent survival between 1986 and 2003: a population study of 5.1 million people. *Circulation.*.

[3] Chen J, Normand SLT, Wang Y, Krumholtz HM (2011). National and Regional Trends in Heart Failure Hospitalization and Mortality Rates for Medicare Beneficiaries, 1998–2008.. JAMA.

[4] Lietz K, Long JW, Kfoury AG, Slaughter MS, Silver MA (2007). Outcomes of left ventricular assist device implantation as destination therapy in the post-REMATCH era: implications for patient selection.. *Circulation*.

[5] Slaughter MS, Rogers JG, Milano CA, Russell SD, Conte JV (2009). Advanced heart failure treated with continuous-flow left ventricular assist device. *N Engl J Med.*.

[6] Gottlieb SS (2009). Prognostic indicators: useful for clinical care? *J Am Coll Cardiol.*.

[7] Kalogeropoulos AP, Georgiopoulou VV, Giamouzis G, Smith AL, Agha SA (2009). Utility of the Seattle Heart Failure Model in patients with advanced heart failure. *J Am Coll Cardiol.*.

[8] Goldraich L, Beck-da-Silva L, Clausell N (2009). Are scores useful in advanced heart failure?. *Expert Rev Cardiovasc Ther*.

[9] Aaronson KD, Schwartz JS, Chen TM, Wong KL, Goin JE (1997). Development and prospective validation of a clinical index to predict survival in ambulatory patients referred for cardiac transplant evaluation.. *Circulation*.

[10] Levy WC, Mozaffarian D, Linker DT, Sutradhar SC, Anker SD (2006). The Seattle Heart Failure Model: prediction of survival in heart failure.. *Circulation*.

[11] Abraham WT, Fonarow GC, Albert NM, Stough WG, Gheorghiade M (2008). Predictors of in-hospital mortality in patients hospitalized for heart failure: insights from the Organized Program to Initiate Lifesaving Treatment in Hospitalized Patients with Heart Failure (OPTIMIZE-HF).. *J Am Coll Cardiol*.

[12] Fonarow GC, Adams KF, Abraham WT, Yancy CW, Boscardin WJ (2005). Risk stratification for in-hospital mortality in acutely decompensated heart failure: classification and regression tree analysis.. *JAMA*.

[13] Martinussem T, Scheike TH (2006). *Dynamic regression models for survival data*.. New York: Springer Verlag.

[14] Aalen OO (1980). A model for nonparametric regression analysis of counting processes.. In: Mathematical statistics and probability theory, editors W. Klonecki, A. Kozek & J. Rosinski. Lecture Notes in Statistics 2: 1–25, New York: Springer-Verlag..

[15] Aalen OO (1989). A linear regression model for the analysis of life times. *Statist. Med.*.

[16] Aalen OO (1993). Further results on the non-parametric linear regression model in survival analysis. *Statist Med.*.

[17] McKeague IW, Sasieni PD (1994). A partly parametric additive risk model.. *Biometrika*.

[18] McKee PA, Castelli WP, McNamara PM, Kannel WB (1971). The natural history of congestive heart failure: the Framingham study. *N Engl J Med.*.

[19] Hunt SA (2005). ACC/AHA 2005 guideline update for the diagnosis and management of chronic heart failure in the adult: a report of the American College of Cardiology/American Heart Association Task Force on Practice Guidelines (Writing Committee to Update the 2001 Guidelines for the Evaluation and Management of Heart Failure). *J Am Coll Cardiol.*.

[20] Kaplan EL Meier P (1958). Nonparametric estimation from incomplete observations.. *Journal of the American Statistical*.

[21] Mantel N (1966). Evaluation of survival data and two new rank order statistics arising in its consideration. *Cancer Chemother Rep.*.

[22] Cox D (1972). Regression models and life-tables. Journal of the Royal Statistical Society Series B (Methodological)..

[23] Cortese G, Scheike TH, Martinussem T (2009). Flexible survival regression modelling.. *Statistical Methods in Medical Research*, 1–24 doi:10.1177/0962280209105022.

[24] Team RDC (2011). R: A language and environment for statistical computing. *R Foundation for Statistical Computing*.. Available at.

[25] Leiden University Medical Center, Department of Medical Statistics and BioInformatics website. *R package coxvc version 1-1-1*.. https://www.msbi.nl/dnn/Research/SurvivalAnalysis/Coxmodelswithtimevaryingeffects.

[26] Grambsch PM, Therneau TM (1994). Proportional hazards tests and diagnostics based on weighted residuals.. *Biometrika*.

[27] Cox DR, Snell EJ (1968). A general definition of residuals. *Journal of the Royal Statistical Society, B*..

[28] Maller R, Zhou X (1996). *Survival analysis with long-term survivals*.. New York:Wiley.

[29] Volinsky CT, Raftery AE (2000). Bayesian information criterion for censored survival models. *Biometrics*..

